# The Medical Association of Georgia

**Published:** 1887-05

**Authors:** 


### THE MEDICAL ASSOCIATION OF GEORGIA.

The thirty-eighth annual meeting of the Medical Association of Georgia, the minutes of which we publish in our present number, deserves to be memorable in the history of that body.

The number of members in attendance was unusually large, the scientific papers presented or read were of marked ability, and the discussion of them exhaustive and earnest. The deep general interest manifested in the entire proceedings evidenced the growing popularity of the Association and the gradual ascent to a higher plane of professional excellence.

The social features of the occasion were at least up to the average, and will leave behind pleasant reminiscences for many years. These little green spots in the dreary desert of a Georgia doctor's life are very precious to his memory. The hope of their annual repetition in coming years will be his encouragement and solace.

A leading feature of the work of the Association was the annual address of the President, Dr. T. O. Powell, Physician to the State Lunatic Asylum, published herewith, to which we respectfully invite public attention. Its philosophy and its practical recommendations are addressed to the General Assembly and to the higher authority—the entire people.

